# Extra Limites

**Published:** 1823-06-01

**Authors:** 


### EXTRA LIMITES.

### MR. LIZARS' REPLY TO A CRITIQUE IN THE MEDICO-CHIRURGICAL REVIEW.

" *While our senses and judgement are in their present imperfect state, it is no wonder that men should differ so widely in their opinions of things, and in the consequences they draw from the appearances in nature.*" MONRO *Primus.*

SINCE the criticism of my Essay on Hernia, with its treatment, in the 10th Number of the Medico-Chirurgical Review, I have received the following letter from Mr. Low, Surgeon, in Queensferry, formerly a patient under this disease. As this gentleman's statement confirms the treatment of hernia, I trust the *contributors* to the Review, will have a little more faith in the doctrine of *tanning* the living body, whether by '*splashing*' or '*bathing*.'

'Queensferry, 27th December, 1822.'

'Dear Sir,'

'The other day I read in the 10th Number of the Medico-Chirurgical Review, some observations upon your paper on the use of oak bark in reducible hernia. I confess I was not surprised at the incredulity of the reviewer, but I felt indignant at the manner in which he treats the subject. It is impossible that our profession can advance while new doctrines or new modes of treatment of diseases are canvassed in such a manner. Be this as it may, I have to call to your recollection, that I was one of your patients who used the oak bark, and I am truly happy to repeat your own words, with '*wonderful success*.' I had been affected with reducible inguinal hernia of my right side for two years, and many a time I was alarmed, when either constipation occurred, or when I failed to reduce it on a short trial. By the application of the oak bark, I have succeeded in completely shutting up the enlarged inguinal aperture, and preventing the intestine descending. I have left off wearing a truss for three years, and I can walk and ride on horseback for miles without the smallest inconvenience. I may say that I ride daily from fifteen to twenty miles, and, occasionally, between twenty and thirty miles.

'Besides its use in hernia, I have found the oak bark decoction equally serviceable in prolapsus uteri. The most marked instance in my practice, is the case of a woman 65 years of age, the mother of fourteen children, who had been subject to this disease after her first child, and excepting during the periods of gestation, she suffered great pain, and was often rendered unable to be of any assistance to her family. Her youngest is now upwards of 18 years old, and for the last twelve years, she had been unable to walk even about the house without suffering excessive pain. When I was called to her in 1818, I found her in bed in great misery. The uterus carrying before it the vagina, descended beyond the labiæ between two and three inches, and the vagina was inflamed and excoriated. She was ordered to foment the parts with warm water until the inflammation should subside, which took place in two or three days. She then began the use of the warm decoction of oak bark, by first reducing the uterus, and injecting with a female syringe the medicine four or five times a day, wearing in the interval, a piece of sponge inserted in the dry state as a pessary, but which was after insertion moistened with the decoction. The sponge at first was about the size of a large apple, but was soon found necessary to be reduced, and when she left off wearing it, it was about the size of a large nut. At each time of injecting the bark, she described her feelings, as 'if it were drawing her together.' She remained the first ten days of the treatment in bed, and in less than three months she was perfectly cured. She has several times since walked about twenty miles a day without the least inconvenience.' (Signed) 'JOHN LOW.'

'*To John Lizars, Esq. Surgeon,*
'39, *St. Andrew Square, Edinburgh.*'

To remove the unbelief further from the minds of the contributors, I beg to refer them to article 28, volume 1, of the Edinburgh Medical Essays and Observations, where they will find the account of a woman labouring under 'hernia inguinalis' cured by the application of 'a strong decoction of oak-bark and alum with strong red wine.' This woman had children afterwards, without any return of the rupture. I have little doubt if all the periodical works were searched, that we should find many cases of this malady cured. It teaches us that there is a fashion in medicines as in every other thing, and that they must fall into disrepute, not according to their own demerits, but according to the ignorance and abuse of those who prescribe them. JOHN LIZARS.

In corroboration of the above statement of the efficacy of a strong decoction of oak-bark in the permanent cure of reducible hernia, the Editor has been informed by his esteemed friend Mr. R. W. Bampfield, that he has, by its use, completely succeeded in the case of a Mr. Thomas Roberts, who had been afflicted with an inguinal hernia about two years, which for some months previous to his application to Mr. B. for advice, had been increasing in size, in consequence of his not having worn a truss. The mode of treatment was rather different from Mr. Lizars's, as Mr. B's patient wore his truss night and day, and used a thicker compress of many folds of linen under the pad of the truss, which he wetted as often as it was becoming dry, without limiting himself to the application of it "three or four times a day only." On the 10th of October, 1822, the use of the decoction commenced, and on the 18th of November, the hernia did not descend even on coughing; the patient, however, continued the use of the decoction about three months.—Mr. B. has seen him lately, and he continues permanently cured. Ed.

